# Effect of P2X_4_ and P2X_7_ receptor antagonism on the pressure diuresis relationship in rats

**DOI:** 10.3389/fphys.2013.00305

**Published:** 2013-10-25

**Authors:** Robert I. Menzies, Robert J. Unwin, Ranjan K. Dash, Daniel A. Beard, Allen W. Cowley Jr., Brian E. Carlson, John J. Mullins, Matthew A. Bailey

**Affiliations:** ^1^University/British Heart Foundation Centre for Cardiovascular Science, The University of EdinburghEdinburgh, UK; ^2^Centre for Nephrology, University College London Medical SchoolLondon, UK; ^3^Department of Physiology, Medical College of WisconsinMilwaukee, WI, USA

**Keywords:** purinergic, ATP, kidney disease, renal injury, renal vascular resistance

## Abstract

Reduced glomerular filtration, hypertension and renal microvascular injury are hallmarks of chronic kidney disease, which has a global prevalence of ~10%. We have shown previously that the Fischer (F344) rat has lower GFR than the Lewis rat, and is more susceptible to renal injury induced by hypertension. In the early stages this injury is limited to the pre-glomerular vasculature. We hypothesized that poor renal hemodynamic function and vulnerability to vascular injury are causally linked and genetically determined. In the present study, normotensive F344 rats had a blunted pressure diuresis relationship, compared with Lewis rats. A kidney microarray was then interrogated using the Endeavour enrichment tool to rank candidate genes for impaired blood pressure control. Two novel candidate genes, *P2rx7* and *P2rx4*, were identified, having a 7− and 3− fold increased expression in F344 rats. Immunohistochemistry localized P2X_4_ and P2X_7_ receptor expression to the endothelium of the pre-glomerular vasculature. Expression of both receptors was also found in the renal tubule; however there was no difference in expression profile between strains. Brilliant Blue G (BBG), a relatively selective P2X_7_ antagonist suitable for use *in vivo*, was administered to both rat strains. In Lewis rats, BBG had no effect on blood pressure, but *increased* renal vascular resistance, consistent with inhibition of some basal vasodilatory tone. In F344 rats BBG caused a significant reduction in blood pressure and a *decrease* in renal vascular resistance, suggesting that P2X_7_ receptor activation may enhance vasoconstrictor tone in this rat strain. BBG also reduced the pressure diuresis threshold in F344 rats, but did not alter its slope. These preliminary findings suggest a physiological and potential pathophysiological role for P2X_7_ in controlling renal and/or systemic vascular function, which could in turn affect susceptibility to hypertension-related kidney damage.

## Introduction

Kidney injury and declining renal function are diagnostic indicators of kidney disease and present a global health burden with high population prevalence (Eckardt et al., 2013). Genetic, epigenetic and environmental factors determine susceptibility to renal injury and the development of chronic kidney disease. Hypertension is a major risk factor for kidney disease (Nakayama et al., 2011) and progression can be slowed if blood pressure is controlled (Hart and Bakris, 2010). Nevertheless, renal injury and fibrosis develop independently of barotrauma and the local actions of agents such as aldosterone (Ashek et al., 2012; Kawarazaki et al., 2012) and angiotensin II (Mori and Cowley, 2004; Polichnowski et al., 2011) have been implicated.

We have previously used the *Cyp1a1-Ren2* transgenic rat to investigate pathways leading to renal injury. In these rats, blood pressure is increased by dietary administration of the non-toxic aryl hydrocarbon, indole-3-carbinol (Kantachuvesiri et al., 2001). The rise in blood pressure can be titrated to study the organ injury associated with slowly developing (Conway et al., 2012) or malignant hypertension (Kantachuvesiri et al., 2001). In the malignant setting, vascular injury predominates, with myocycte vacuolation preceding confluent myocyte cell death and microalbuminuria (Ashek et al., 2012).

Genetic background influences susceptibility to renal injury in several rat models (Churchill et al., 1997; Schulz and Kreutz, 2012), the *Cyp1a1-Ren2* transgenic rat being no exception (Kantachuvesiri et al., 2001). Here, the Fischer (F344) strain is susceptible while *Cyp1a1-Ren2* transgenic rats on the Lewis background are protected from renal injury. We have used these informative strains to identify Quantitative Trait Loci for organ injury (Kantachuvesiri et al., 1999) and the development of reciprocal congenic lines enabled us to validate *Ace*, the gene encoding Angiotensin Converting Enzyme, as a plausible modifier of renal injury (Liu et al., 2009). Although the angiotensin receptor antagonist losartan prevents the blood pressure rise in this model, it is only partially protective against renal vascular injury (Ashek et al., 2012). This suggests that susceptibility to renal injury in this model is governed by the interplay between multiple pathways. We hypothesized that genes differentially expressed in the *Cyp1a1-Ren2* transgenic rat in the normotensive state would contain candidates contributing to poor renal function and susceptibility to renal injury in the F344 strain or the relative renoprotection observed on the Lewis background.

In the present study we compared the pressure diuresis relationship between the differentially susceptible F344 and Lewis rats. This response being blunted in F344 animals, we re-mined a renal exon-microarray (Liu et al., 2009) identifying the genes encoding the P2X_4_ receptor and P2X_7_ receptor as candidates for altered vascular function in F344 rats.

## Materials and methods

### Microarray analysis

A previously published Affymetrix microarray (Liu et al., 2009) was re-mined to identify differentially expressed probe-sets in the kidney of normotensive *Cyp1a1-Ren2* transgenic rats, i.e., rats in which the *Ren2* transgene was silent. The array was performed on four groups of rats (*n* = 4 per group): the two consomic parental strains (F344, Lewis) and the two reciprocal congenic strains (F344-MOD-Lewis, Lewis-MOD-F344) containing a 14 Mb region of chromosome 10. This congenic region contained the *Ace* locus and the congenics were included in the present analysis to determine whether cis (or trans) regulation occurred. The 16 CEL intensity files were imported into Bioconductor and arrays normalized by the Robust Multi-array Average (RMA) method. The Linear Models for Microarray Data (LIMMA) algorithm was used to calculate fold-change and *p*-value statistics from the normalized intensities.

Differentially expressed genes were imported into the web client online version of the multi-database enrichment tool Endeavour (Aerts et al., 2006, 2009). A list of 157 “training” genes isolated from the rat genome database (Laulederkind et al., 2002) was also imported. The “training” genes used in this study were selected for their association with blood pressure regulation in the rat. They were not tissue specific and assumed no mutual exclusivity with inflammatory, or other disease, processes. The Endeavour method then employed multiple database mining using parallel approaches to enrich the list of differentially regulated genes. These approaches were: (i) published literature text mining; (ii) protein-protein interactions in the STRING database; (iii) transcriptome analysis from the WalkerEtAl database; (iv) sequence comparison with BLAST; and (v) annotations within Gene Ontology, InterPro, KEGG, and Swiss-Prot. Finally, global ranking by Q-statistic generated a list of genes in order of prioritization for the observed phenotype, known as “genomic data fusion.”

### Animals

*E*xperiments were performed on male F344 and Lewis rats (Charles River, UK). All rats had access to food and water (Special Diet Services, Witham, Essex, UK) *ad libitum*. Procedures were performed in accordance with the UK Home Office Animals (Scientific Procedures) Act of 1986 after ethical review by The University of Edinburgh.

For Western analysis and immunohistochemistry, F344 and Lewis rats (*n* = 3 per genotype) were killed by decapitation. The kidneys were rapidly excised and the left kidney was snap frozen and stored at −80C for subsequent extraction of total protein. The right kidney was immersion fixed in 10% buffered formalin, transferring to 70% ethanol after 48 h. These kidneys were then paraffin embedded and transverse sections taken for IHC.

### Immunohistochemistry

Primary rabbit polyclonal antibodies against the P2X_1_ (APR-001, Alomone Labs), P2X_4_ (APR-002, Alomone Labs), and P2X_7_ (APR-004, Alomone Labs) receptors were selected based on published validation for use in the rat. Each antibody was then optimized in a dilution series (1:250, 500, 1000, 2000, 4000, 5000, and 7500) using control rat kidney, following heat-induced epitope recovery (HIER) with citrate buffer. The final titers were selected to give minimal background: P2X_1_ (1:5000), P2X_4_ (1:7500), and P2X_7_ (1:2000). All staining was performed on a Leica Bond × immunostaining robot using a refined HRP polymer detection system. Briefly, after HIER and blocking in Peroxidase, the section was incubated in primary antibody for 2 h at room temperature. Following two 5 min washes, sections were exposed to anti-rabbit HRP polymer before being washed. Immunopositive staining was visualized with 3,3′-diaminobenzidine and counterstaining with hematoxylin.

### Western blot

Whole kidneys were homogenized in ice-cold buffer containing 250 mmol/l sucrose and 10 mmol/l triethanolamine. Protease inhibitors (Cocktail set III, Calbiochem) and phosphatase/kinase inhibitors (2 mmol/l EDTA, 50 mmol/l NaF, 25 mmol/l sodium glycerophosphate, 5 mmol/l pyrophosphate, and 1 mmol/l sodium orthovanadate) were added and the pH adjusted to 7.6. Following quantification by Bradford assay, protein samples were added to Laemlli buffer and resolved by SDS-PAGE, on a NuPAGE Tris-Acetate gel (8% NovexTM) using a Tris-acetate running buffer (50 mmol/l tricine, 50 mmol/l Tris base, 0.1% SDS, pH 8.24) NuPAGE antioxidant was added to the upper chamber. For the P2X_4_ studies, 12 μg of total protein was loaded; 20 μg for P2X_7_ receptor experiments. Following semi-dry transfer the membrane was incubated overnight at 4C with the primary antibody (P2X_4_ 1:2000; P2X_7_ 1:1000; Alomone as described above). A goat-antirabbit HRP secondary antibody was then added and the bands visualized by ECL. The P2X_4_ antibody detected a band of ~60 kDa; the P2X_7_ antibody detect a band at ~75 kDa. The autoradiogram was scanned and band intensity (corrected for background) was quantified by densitometry using ImageJ. Values were normalized to the total protein intensity (Coomasie-Blue) at the appropriate molecular weight.

### Renal functional studies

Rats were anaesthetized (Thiobutabarbital 120 mg/kg IP) and prepared surgically for measurement of the pressure-diuresis relationship. The right jugular vein was cannulated and 0.9% NaCl was infused at a rate of 50 μ l/min/100 g during abdominal surgery (to replace surgical losses) and then at 33 μ l/min/100 g during the post-surgical equilibration (60 min) and throughout the experimental protocol. The left femoral artery was cannulated and connected to brass transducer (MLT844; Capto) connected to a Powerlab (AD Instruments, UK). Blood pressure was recorded continuously at 1 kHz. A midline laparotomy was performed and a Doppler transit time probe (MA1PRB; Transonic, USA) placed around the left renal artery. Acoustic gel was used to ensure good sonic coupling. Loose silk ties were placed around the superior mesenteric and coeliac arteries: these ligatures were tightened during the experimental procedure to create an acute pressure ramp of two stages above baseline blood pressure. The bladder was catheterized for urine collection under mineral oil with flow rate being determined gravimetrically. The entire procedure was performed under homeostatic temperature control at 37°C.

Pressure-diuresis experiments were performed first on a control group of F344 (*n* = 7) and Lewis (*n* = 5) rats and then on a second cohort of F344 (*n* = 5) and Lewis (*n* = 6) rats receiving an IV infusion (50 μ g/min/100 g) of Brilliant Blue G (BBG, Sigma, UK).

### Statistical analysis

Data are presented as mean ± s.e.m. or as individual data with median. Statistical analysis was performed by Mann-Whitney *U*-test (for Western analysis) or by unpaired *t*-test (physiological data). Comparisons between groups of the pressure-diuresis relationship were made by linear regression.

## Results

### Pressure diuresis relationship

**Figure 1:**
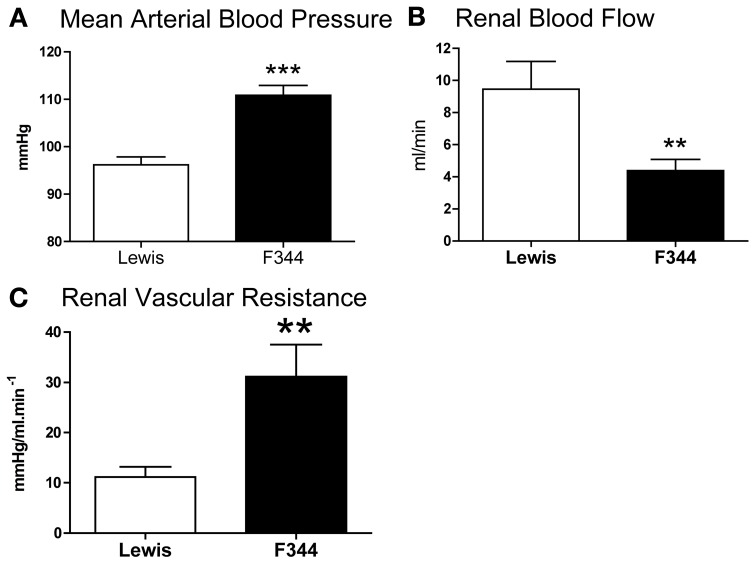
**(A)** Mean arterial blood pressure; **(B)** left renal artery blood flow and **(C)** renal vascular resistance in the left renal artery measured in Lewis (*n* = 8; open bars) and F344 (*n* = 7; black bars) rats. Data are mean ± *SE*. Statistical comparisons were made with unpaired *t*-test. ^***^P < 0.001; ^**^P < 0.01.

**Figure 2:**
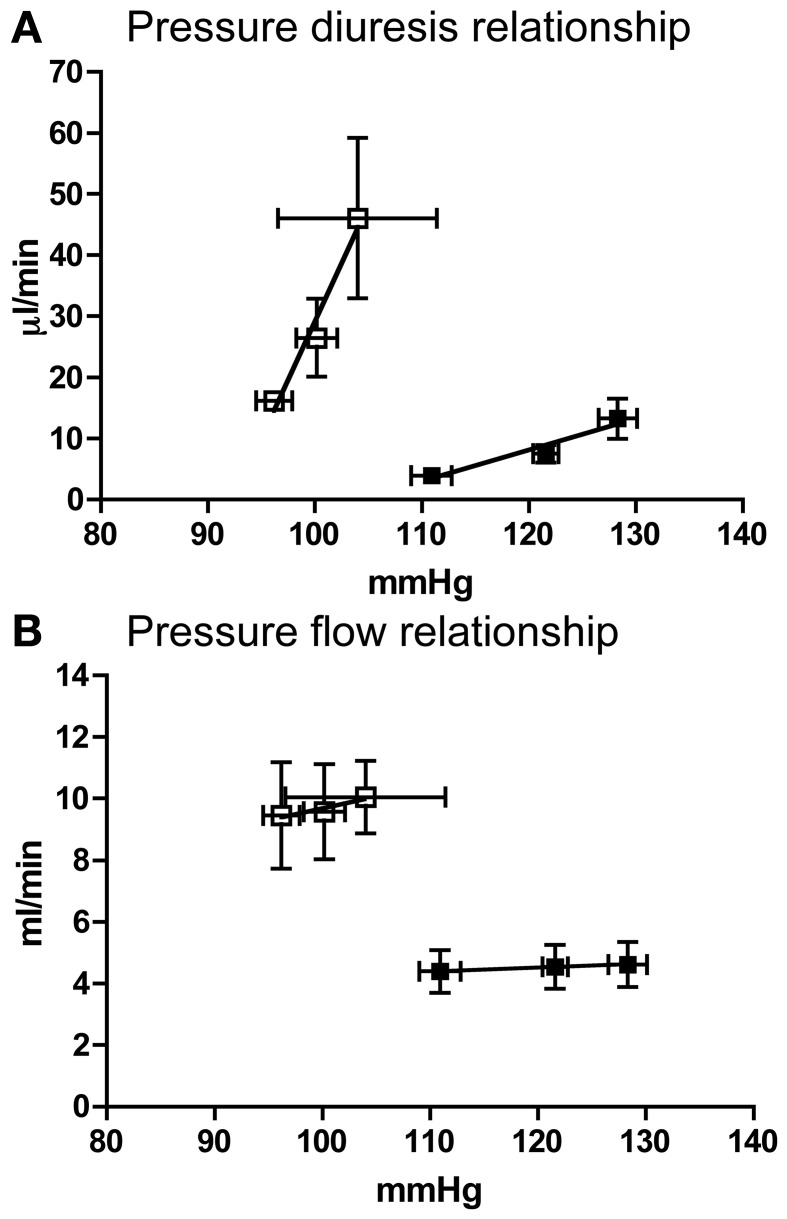
**(A)** Pressure diuresis and **(B)** pressure flow relationship in Lewis (*n* = 8; open squares) and F344 (*n* = 7; black squares) rats. Data are mean ± *SE*. Statistical test was performed by linear regression analysis.

Compared to Lewis rats, F344 rats had a higher baseline blood pressure (Figure 1A) and a lower renal blood flow (Figure 1B): renal vascular resistance was significantly higher in F344 rats than in Lewis (Figure 1C). The imposition of a pressure ramp evoked an increase in urine flow rate in both strains of rats (Figure 2A). The slope of the relationship was significantly different from zero in both groups (*P* < 0.001) but was blunted in the F344 strain compared to the Lewis (*P* < 0.01). There was no significant relationship between blood flow and blood pressure in either strain of animals, indicative of intact auto-regulation (Figure 2B).

### Renal microarray analysis

**Figure 3:**
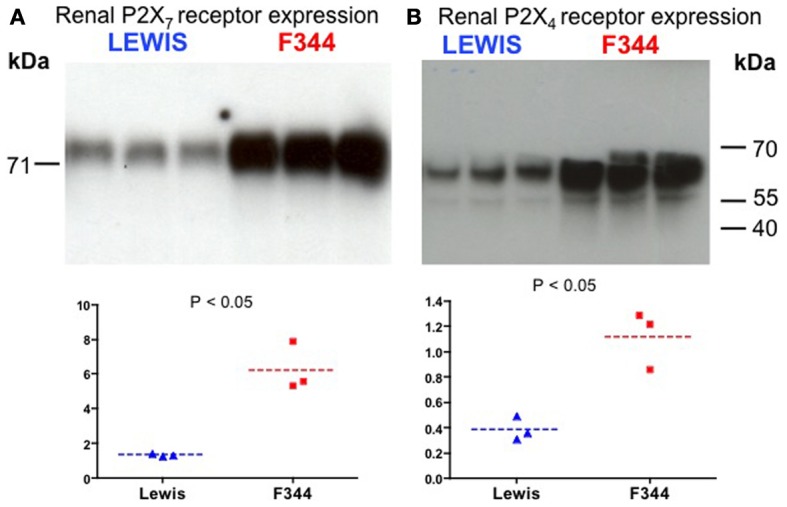
**Western blot analysis of (A) P2X_7_ receptor and (B) P2X4 receptor expression in whole kidney homogenates.** The top panels show the blot performed in Lewis and F344 rats (*n* = 3 in each). The bottom panels show the blot intensity (normalized to protein loading) quantified by densitometry.

After normalization, 67 probe-sets were differentially regulated on the basis of genetic background: 23 over-expressed and 44 under-expressed (Table 1). Endeavour analysis was used to rank the differentially expressed genes enriched against the training genes of blood pressure regulation. The ten highest globally ranked genes are given in Table 2. *Ace* was the highest ranked gene, consistent with our previous QTL and congenic studies (Liu et al., 2009), and was not studied further. The 2nd and 3rd ranked genes were *P2rx7* and *P2rx4*, respectively. The expression of both was higher in the F344 rats than in the Lewis rats. This was confirmed by Western analysis: there was a 7-fold increase in total P2X_7_ receptor protein (*P* < 0.05; Figure 3A) and a 3-fold increase in P2X_4_ receptor abundance (*P* < 0.05; Figure 3B).

**Table 1:** **Genome wide comparison of gene expression between F344 and Lewis inbred strains listed in order of magnitude of fold change (F344 vs. Lewis, fold > ± 1.2, *p* < 0.05)**.

**Over expressed genes (+)**			**Under expressed genes (−)**		
**Symbol**	**Fold**	***p*-value**	**Symbol**	**Fold**	***p*-value**
*Rpl30*	+7.6798	0.0226	*Olr1668*	−27.2451	0.0123
*Akr1c2*	+7.3466	0.0241	*Olr1680*	−24.6268	0.0162
*Spta1*	+5.6906	0.0090	*RGD1309362*	−13.1217	0.0162
*Akr1b8*	+4.6613	0.0178	***Pigzl1***	**−6.7012**	**0.007**
*LOC361914*	+3.6785	0.0094	***Kif5c***	**−6.6248**	**0.009**
***Ace***	**+3.5400**	**0.0178**	*Ces1e*	−5.7903	0.0094
*LOC100359585*	+3.3860	0.0250	*Cyp4v3*	−5.2337	0.0166
*Guca2b*	+2.7994	0.0479	*Olr1326*	−5.1722	0.0336
*Ypel4*	+2.7596	0.0253	*Acsm5*	−4.7035	0.0178
*Rtp4*	+2.6916	0.0241	*Hhip*	−4.6118	0.0166
*Clstn2*	+2.5879	0.0253	*Hmgcs2*	−4.2039	0.0336
***P2rx4***	**+2.5327**	**0.0162**	*Cyp2d5*	−3.8624	0.0289
***Klkb1***	**+2.4303**	**0.0090**	***Rdh2***	**−3.4214**	**0.0162**
*Exnef*	+2.4073	0.0090	*LOC302192*	−3.3622	0.0256
***Pigr***	**+2.3473**	**0.0336**	*Lcn2*	−3.097	0.0253
***P2rx7***	**+2.1586**	**0.0336**	*Csmd1*	−3.019	0.0336
*Akr1b7*	+2.1071	0.0336	*Slc10a2*	−2.7769	0.0226
*Cd59*	+1.8540	0.0256	*Rxrg*	−2.6987	0.0336
*Fam149a*	+1.7008	0.0336	*Cntnap4*	−2.6686	0.0192
*P4ha2*	+1.6668	0.0336	*RT1-CE5*	−2.6679	0.0336
*Arl4d*	+1.5187	0.0336	***Erc2***	**−2.5297**	**0.0253**
*Igfbp4*	+1.4873	0.0336	*Ptprq*	−2.4522	0.0182
*Col15a1*	+1.2734	0.0336	*RGD1311723*	−2.4244	0.0372
			*Rbp4*	−2.3816	0.0336
			*Abcb10*	−2.2669	0.0256
			*Sult1b1*	−2.2336	0.0493
			*RGD1563120*	−2.1689	0.045
			*Mis18a*	−2.1532	0.0192
			*Slc35f1*	−2.1291	0.0372
			*Tcerg1l*	−2.0443	0.0253
			*Acadsb*	−1.9181	0.0336
			***Rgs7***	**−1.8925**	**0.0277**
			*Retsat*	−1.8721	0.0253
			*Gas2*	−1.8114	0.045
			*Ly75*	−1.74	0.0442
			*Slco1a6*	−1.7194	0.031
			*Slc26a11*	−1.6736	0.0317
			*Pfas*	−1.6633	0.0178
			*Eps8l2*	−1.6505	0.0336
			***Dpp6***	**−1.6382**	**0.0259**
			*RGD1311575*	−1.5914	0.0491
			*RGD1564614*	−1.5199	0.0344
			*Cdc42ep2*	−1.4477	0.0372
			*Synm*	−1.4011	0.0442

Genes identified by enrichment analysis (Table 2) shown in bold font.

**Table 2:** **Global prioritization by the Endeavour enrichment method**.

**Gene**	**Known biological function(s)**	**Global prioritization**		
		**Rank**	**Score**	**Rank ratio**
*Ace* (ENSRNOG00000007467)	BP regulation	1	0.0187	0.0909
*P2rx7* (ENSRNOG00000001296)	Ion transport, cell volume, apoptosis	2	0.0624	0.182
*P2rx4* (ENSRNOG00000001300)	Ion transport, BP regulation, NOS	3	0.118	0.273
*Rgs7* (ENSRNOG00000021984)	G-protein signaling	4	0.583	0.364
*Erc2* (ENSRNOG00000015148)	Nerve terminal assembly	5	0.674	0.455
*Klkb1* (ENSRNOG00000014118)	Proteolysis, coagulation, inflammation	6	0.787	0.545
*Kif5c* (ENSRNOG00000004680)	Motor axon guidance	7	0.796	0.636
*Dpp6* (ENSRNOG00000030547)	Proteolysis	8	0.933	0.727
*Pigr* (ENSRNOG00000004405)	Antibody receptor	9	0.936	0.818
*Rdh2* (ENSRNOG00000029651)	Retinoid metabolism, oxidation reduction	10	0.988	0.909

### Renal localization of P2X_1,4, and_ 7 receptors

**Figure 4:**
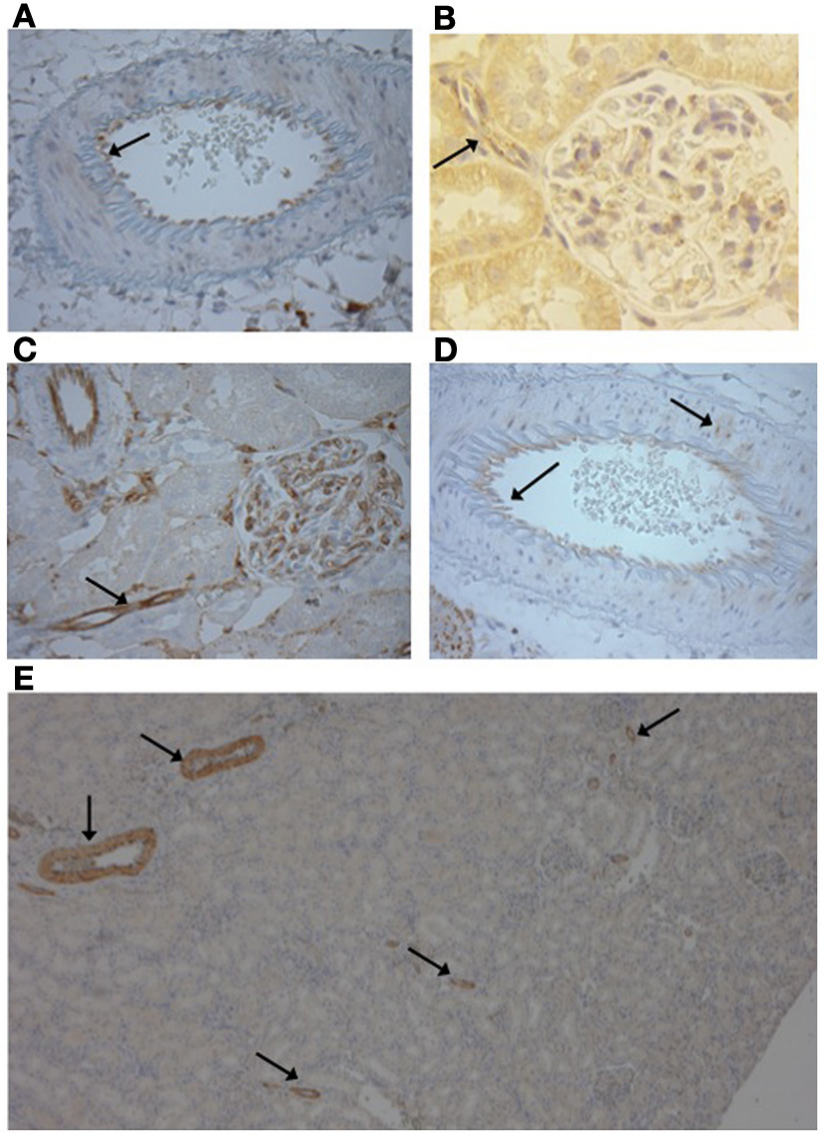
**(A)** Vascular P2X_4_ receptors were expressed in the endothelium (Image from F344 rat ×400) and **(B)** the afferent arteriole (Image Lewis rat ×500). **(C)** P2X_7_ receptors were stained in the endothelium of the preglomerular vasculature, including the afferent arteriole (arrow) and cells of the glomerulus (Image F344 rat, ×400). **(D)** Occasional smooth muscle staining of P2X_7_ was observed (arrow; Lewis rat, ×400). **(E)** P2X_1_ immunopositive staining was only observed in the vasculature and was limited to the smooth muscle layer of large and small diameter vessels (F344 rat, ×50).

We observed no differences between strains in the distribution of immunostaining for the P2X receptors. Renal vascular P2X_4_ immuno-positive staining was restricted to the endothelium throughout the preglomerular vasculature (Figure 4A). P2X_4_ receptor staining was observed in the renal tubules of both strains. In some places this staining was punctate and localized to both the nucleus and cytoplasm (Figure 4B).

Vascular P2X_7_ receptor staining was observed in the endothelium of the pre-glomerular arteries, including the afferent arterioles of both rat strains (Figures 4B,C). Staining was also observed in the glomerulus (Figure 4C). In the larger arteries, occasional expression in the vascular smooth muscle was observed but in a given vessel this was limited to a small number of myocytes (Figure 4D).

As shown by the low magnification image, P2X_1_ receptor expression was limited to the vascular network and not expressed in the renal tubules (Figure 4E). P2X_1_ receptor immuno-positive staining was observed in the smooth muscle layer of all artery types from lobar to afferent arteriole in both rat strains.

### Effect of infusion of brilliant blue g

**Figure 5:**
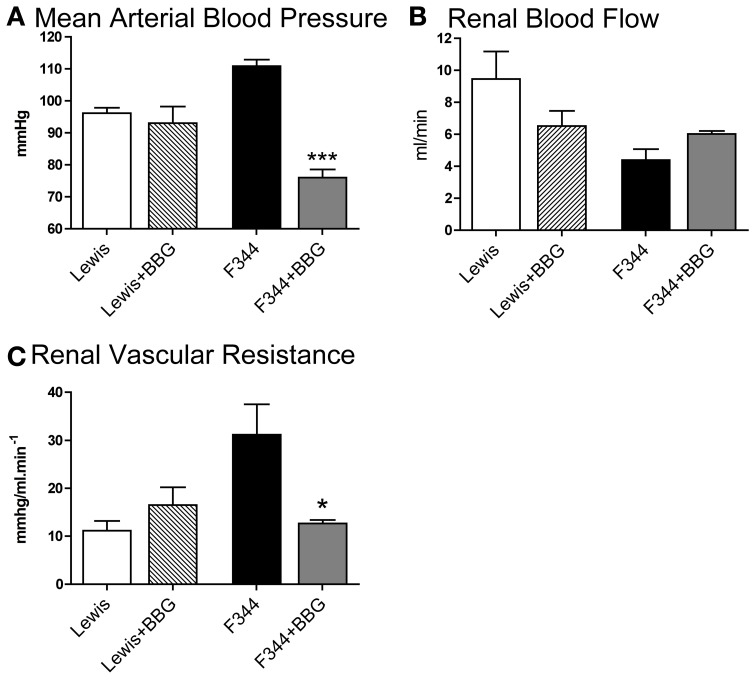
**(A)** Mean arterial blood pressure; **(B)** left renal artery blood flow and **(C)** renal vascular resistance in the left renal artery measured in Lewis and F344 rats receiving either saline or Brilliant Blue G by intravenous infusion. Data are mean ± *SE*. Statistical comparisons were made within strain by unpaired *t*-test. ^***^P < 0.001; ^*^P < 0.05. Statistical comparisons were made using one way ANOVA with Bonferroni post-test.

**Figure 6:**
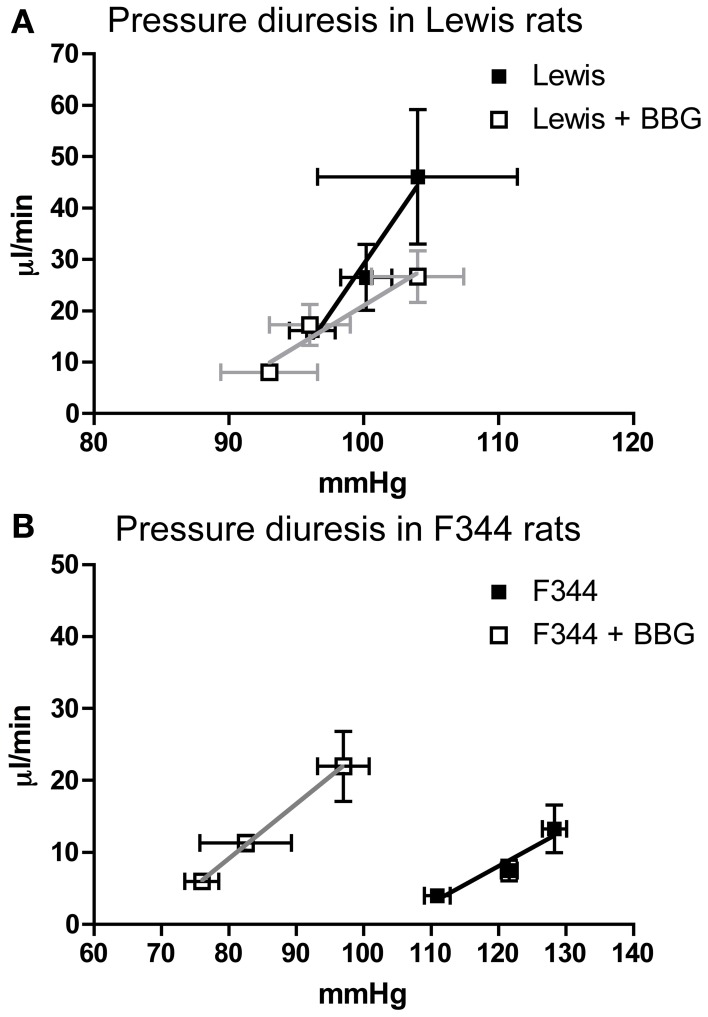
**The Pressure diuresis relationship measured in (A) Lewis and (B) F344 rats receiving either saline (closed symbols) or Brilliant Blue G (open symbols) by intravenous infusion.** Data are mean ± *SE*. Statistical test was performed by linear regression analysis.

Under baseline (non-ligated) conditions, acute infusion of BBG caused a significant reduction of mean arterial blood pressure in F344 rats but not in Lewis animals (Figure 5A). Blood flow through the left renal artery was not significantly affected by BBG in either group (Figure 5B). However, BBG caused a significant *decrease* in renal vascular resistance in F344 rats (Figures 5C, 6B).

Acute infusion of BBG did not affect the pressure-diuresis relationship in Lewis rats (Figure 6A). In F344 rats, BBG caused a significant leftward shift of the pressure-diuresis intercept (Figure 6B), reducing the threshold of this response, but did not alter the gradient of the slope. There was no significant relationship between blood flow and blood pressure in either strain (data not shown).

## Discussion

F344 rats are susceptible to renal vascular injury whereas Lewis rats are relatively protected (Liu et al., 2009). We find that normotensive F344 rats have a blunted pressure diuresis relationship, which would impair blood pressure control and may underpin the susceptibility to vascular injury observed in this strain. At a genetic level, we identified increased renal expression of P2X_4_ and P2X_7_ receptors, which may contribute to impaired vascular function in F344 rats, compared to the Lewis strain.

Multiple subtypes of P2X and P2Y receptors are expressed throughout the kidney and extracellular nucleotides regulate renal tubular, endocrine, and vascular functions (Bailey and Shirley, 2009; Bailey et al., 2012; Shirley et al., 2013). Purinergic control of renal vascular tone is complex and the net vasoactive effect depends upon the route of administration/physiological source of the extracellular nucleotide. Thus, ATP applied *in vitro* to the adventitial surface of the renal microvasculature causes contraction (Inscho et al., 1992) mediated by P2X_1_ receptors (Inscho et al., 2003) in the vascular smooth muscle (Chan et al., 1998). In contrast, infusion of ATP into the renal artery increases blood flow (Tagawa and Vander, 1970) and the vasodilatation is dependent on production of nitric oxide/prostacyclin by the endothelium (Eltze and Ullrich, 1996). The P2 receptor subtype(s) that mediate the vasodilatory response to ATP is not resolved and may vary in different vascular beds. mRNA encoding P2Y_1_, P2Y_2_, P2X_4_, and P2X_7_ receptors have all been identified in human arterial endothelial cells (Yamamoto et al., 2000; Ray et al., 2002). P2X_4_ receptors are the most abundantly expressed, followed by P2X_7_ (~50%) and then by P2Y_1_ and P2Y_2_ (~20%) receptors (Yamamoto et al., 2000). A similar profile is observed in endothelial cells cultured from the mouse pulmonary artery (Yamamoto et al., 2006) and P2X_4_ and P2X_7_ receptors have also been immunolocalized to the endothelium of the larger renal arteries of the rat (Lewis and Evans, 2001).

Our studies are largely consistent with this distribution of P2X receptors. P2X_1_ receptor expression was limited to the vascular smooth muscle of the renal arteries and afferent arteriole. Renal autoregulation is severely attenuated in P2X_1_ null mice, (Inscho et al., 2004; Guan et al., 2007; Inscho, 2009), illustrating the importance of this receptor for renal vascular function. In the present study, renal autoregulation was intact in both strain of rats and we find no evidence linking differential expression of the P2X_1_ receptor, or indeed P2X4 or P2X7 receptors to the impaired renal vascular function observed in F344 rats.

We did find increased abundance of P2X_4_ and P2X_7_ receptor, both in the microarray analysis and at the protein level. In humans the encoding genes, *P2RX4* and *P2RX7*, are located within 130 kb of each other on chromosome 12. These genes can be regulated independently: the endothelial expression of P2X_4_ receptors in the human aorta is increased following injury; P2X_7_ receptor expression is not affected (Pulvirenti et al., 2000). It is possible, however, that these receptors have common promotor elements. Physiological interactions between the receptors are postulated (Craigie et al., 2013) and the locus is associated with human disease. For example, a single nucleotide polymorphism (SNP) in the first intron of *P2RX7* is strongly associated with elevated blood pressure (Palomino-Doza et al., 2008) and a loss-of-function SNP in the *P2RX7* coding region associates with protection against ischemic stroke (Gidlöf et al., 2012). Similarly, a loss of function SNP in the P2X_4_ receptor has been associated with increased pulse pressure (Stokes et al., 2011).

Consistent with the previous studies described, we localized P2X_4_ and P2X_7_ receptors to the endothelium of the pre-glomerular vasculature. Our bioinformatic ranking analysis associated increased expression with vascular dysfunction and loss of blood pressure control. Both P2X_4_ (Yamamoto et al., 2006) and P2X_7_ receptors (Liu et al., 2004) can modulate blood vessel contractility by promoting the release of vasodilators from the endothelium. One interpretation of our data is that the up-regulation of receptors in F344 rats is a compensatory response to improve poor renal blood flow. Thus, acute receptor antagonism *in vivo* should inhibit this tonic vasodilation. There was a trend for this in the Lewis rats but the reduction in blood flow induced by BBG was not statistically different. BBG did induce a significant hemodynamic effect in F344 rats but this was to increase blood flow, rather than to reduce it. One interpretation of this outcome is that in F344 rats P2X_4_/P2X_7_ receptor activation induces a tonic vasoconstriction. It is difficult to reconcile such an effect with the predominantly endothelial location of these receptors. However, the endothelium also releases potent vasoconstrictive mediators, including mono- or di-nucleoside polyphosphates such as adenosine 5′ tetraphosphate (Tolle et al., 2008) and uridine adenosine tetraphosphate is a partial agonist at the rat P2X_4_ receptor (Wildman et al., 1999) and causes a profound vasoconstriction when perfused via the intravascular route into the isolated rat kidney (Tolle et al., 2008).

An obvious concern in interpreting these results is the selectivity of the antagonist, BBG. This compound is a potent inhibitor of rat P2X_7_ receptors (IC_50_= 10 nM) and although it can also block the P2X_4_ receptor, its selectivity for P2X_7_ receptor is 1000-fold greater. BBG has been used previously *in vivo* to elucidate P2X_7_ receptor functionality (Jiang et al., 2000; Peng et al., 2009). Indeed, chronic administration of BBG reduces renal injury and lowers blood pressure in the Dahl salt sensitive rat (Ji et al., 2012a); P2X_7_ null mice are similarly protected from the renal injury associated with salt-induced hypertension (Ji et al., 2012b). Nevertheless, BBG may also antagonize rat P2X_4_ receptors and our infusion protocol could inhibit both P2X receptor subtypes. Furthermore, a number of off-target effects of BBG have been reported (Katrahalli et al., 2010), so we cannot exclude the possibility that P2X_7_-independent effects also contribute to the hemodynamic actions of BBG observed in the F344 rats.

P2X_4_ and P2X_7_ receptors were also identified in the renal tubule in both strains of rats. Tubular expression of P2X_4_ receptor is consistent with several previous studies (Bailey et al., 2012). We found some evidence of intracellular, punctate staining, particularly in the Lewis rats. It is possible that this represents expression of P2X_4_ receptors in intracellular vesicles, which might act as a reservoir for trafficking of receptors to the apical or basolateral membrane or serve as mediators of vacuolar calcium release (Sivaramakrishnan and Fountain, 2012). P2X receptors, including P2X_4_ can regulate tubular sodium reabsorption processes (Bailey et al., 2012) but in our studies BBG did not affect urine flow rate. The relationship between P2X_4_ receptor activation and sodium/water reabsorption is complex, however, and may depend on the local sodium concentration.

In summary, P2X_7_ and P2X_4_ receptors are expressed in the vascular endothelium and may contribute to the normal control of renal arterial resistance. Both receptors are attractive candidate genes for impaired renal vascular function and susceptibility to kidney injury. However, their respective roles are not easy to define: the present findings are consistent with a predominant vasoconstrictor effect of P2X_7_ and vasodilator effect of P2X_4_, but the relationship is likely to be more complex than this simple dichotomy suggests. For example, endothelial P2X_7_ receptors can mediate the release of factors that modulate the inflammatory state of the vessel wall (Wilson et al., 2007). Moreover, the encoding gene for P2X_7_ transcribes a large number of splice variants with reportedly different functionality (Sluyter and Stokes, 2011; Xu et al., 2012), which may also contribute to contrasting vasoactive effects in different strains of rat as observed here.

## Author contributions

Performing experiments: Robert I. Menzies, Data analysis: Robert I. Menzies, Matthew A. Bailey, Data interpretation: Robert I. Menzies, John J. Mullins, Robert J. Unwin, Matthew A. Bailey, Discussion of data and manuscript: all authors, Writing of paper: all authors

### Conflict of interest statement

The authors declare that the research was conducted in the absence of any commercial or financial relationships that could be construed as a potential conflict of interest.
